# Injectable and Self-Healing Boronic-Acid-Modified Succinoglycan Hydrogels: Dual-Stimuli-Responsive Platforms for Controlled Tannic Acid Release

**DOI:** 10.3390/gels11110897

**Published:** 2025-11-09

**Authors:** Eunkyung Oh, Jae-pil Jeong, Sobin Jeon, Seunho Jung

**Affiliations:** 1 Department of Bioscience and Biotechnology, Microbial Carbohydrate Resource Bank (MCRB), Konkuk University, 120 Neungdong-ro, Gwangjin-gu, Seoul 05029, Republic of Korea; eunkyung_5@naver.com (E.O.); jjp0531@naver.com (J.-p.J.); 2 Department of System Biotechnology, Microbial Carbohydrate Resource Bank (MCRB), Konkuk University, 120 Neungdong-ro, Gwangjin-gu, Seoul 05029, Republic of Korea; wjsthqls030414@naver.com

**Keywords:** succinoglycan, phenylboronic acid, tannic acid, hydrogel, antibacterial activity, antioxidant activity, dual-responsive

## Abstract

In this study, succinoglycan (SG), an anionic exopolysaccharide derived from *Sinorhizobium meliloti* Rm1021, was chemically modified to introduce boronic acid groups, creating a boronic-acid-functionalized polysaccharide (SG-APBA). The degree of substitution varied from 4.24% to 24.3%, depending on APBA concentration, with SG-APBA 2 identified as the optimal formulation. The properties of SG-APBA were characterized using ^1^H NMR, FTIR, TGA, and XRD, along with rheological analysis to assess changes in the polymer’s behavior. The hydrogel, referred to as SAT, was formed through dynamic boronate-ester bonds and hydrogen bonds between SG-APBA and tannic acid (TA). This hydrogel demonstrated excellent injectability, self-healing capacity, and biocompatibility. Incorporation of boronic acid groups allowed the hydrogel to respond to variations in glucose levels and pH, enabling controlled TA release and enhancing its stimulus-responsive antioxidant and antibacterial activities. Antioxidant performance was confirmed through DPPH and ABTS radical scavenging assays, achieving respective activities of 89.8% and 96.4%. Antibacterial effectiveness was validated via inhibition zone tests. Additionally, the SAT hydrogel exhibited dual responsiveness to pH and glucose, with TA release percentages of 55.4% at pH 9.0, 62.7% at pH 7.4, and 69.9% at pH 5.0; and 62.7% at 0 mM glucose, 68.9% at 5 mM, and 72.5% at 25 mM glucose after 120 h. Moreover, combined alterations in pH and glucose triggered a synergistic double-shock effect, markedly accelerating TA release relative to individual stimuli. Overall, these results indicate that the SG-APBA/TA hydrogel has strong potential as a stimuli-responsive platform for drug delivery and biomedical applications.

## 1. Introduction

Hydrogels are generated through chemical or physical cross-linking of hydrophilic polymers, resulting in a three-dimensional network structure. Notably, they have the ability to hold significant amounts of water and expand in size without disintegrating [1,2,3]. Hydrogels can encapsulate drugs within their network and offer diverse release mechanisms depending on the polymer type, allowing for the delivery of various therapeutic agents such as peptides, nucleic acids and natural products [4,5,6]. Based on these advantages, previous studies have identified hydrogels as versatile platforms for various biomedical applications, including wound healing and drug delivery [7,8]. Hydrogels can be constructed from either synthetic or naturally derived polymers. Among natural polymers, polysaccharides are particularly notable for forming hydrogels tailored to specific needs based on their molecular weight, functionality, and polarity [7,9]. Due to their high biocompatibility, non-toxicity, and biodegradability, polysaccharides are widely used in hydrogel fabrication [10].

Succinoglycan (SG) is a microbial polysaccharide historically used as an emulsifier and thickening agent [11]. Its structure comprises a repeating octasaccharide unit with non-carbohydrate moieties, including pyruvic acid, succinate, and acetate, and features a glucose to galactose molar ratio of 7:1 [12]. Recent research has revealed a variety of new functionalities, including anticancer, antibacterial, and anti-inflammatory activities [13,14,15]. Based on these properties, SG is being actively utilized as a material for hydrogels, films, and other applications [16,17,18]. Furthermore, ongoing studies focus on chemically modifying SG to enhance or introduce additional properties such as viscosity, antibacterial, and antioxidant activities [19,20].

Also, extensive research efforts focus on developing functional hydrogels for enhanced drug delivery applications, with particular emphasis on stimuli-responsive systems in the past few decades [21]. For example, wounds in diabetic patients are exposed to higher pH and glucose levels compared to normal skin. Therefore, it is essential to develop hydrogels tailored to these conditions to ensure effective drug delivery [22]. Many researchers have utilized phenylboronic acid (PBA) as a versatile building block for designing responsive materials. PBA is capable of undergoing reactions with functional groups in response to variations in pH, glucose concentration, and the presence of reactive oxygen species (ROS) [23]. Among dynamic covalent bonds, boronic acid esters are increasingly favored due to their high chemical reactivity, occurrence under mild conditions, and the ability to form reactions without the need for catalysts [24]. PBA modification involves attaching PBA groups to a wide range of materials, such as nanoparticles, polymers, and hydrogels [25,26,27]. This process can improve the functionality of material, for example, by increasing its affinity for certain biological molecules or boosting its therapeutic performance. By applying PBA incorporation techniques to polysaccharides, numerous examples of their integration into the biomedical field have demonstrated exceptional functionalities, as evidenced by various studies [28,29].

To improve gelation efficiency and boost functionality, natural additives are occasionally added to polysaccharide-PBA derivatives, and tannic acid is one of the prominent examples [30,31,32]. Tannic acid is a naturally occurring polyphenol composed of a central glucose molecule esterified with several galloyl groups. The number of galloyl units can differ, generally ranging from 2 to 12, but the commonly recognized structure of tannic acid typically features around 10 galloyl groups [33]. Tannic acid can enhance the stability of hydrogels through interactions between its galloyl groups and PBA [34]. Additionally, its intrinsic antibacterial, anti-inflammatory, and anticancer properties can impart strong functionalities to the hydrogel [35,36,37]. Moreover, its reactivity with metals can be harnessed to confer various stimuli-responsive behaviors [38].

In this study, 3-aminophenylboronic acid (3-APBA) was successfully grafted onto SG through DMTMM-mediated amidation of succinyl and pyruvyl groups. Nowadays, DMTMM is used as a coupling agent because of its higher yield and amidation efficiency compared with 1-ethyl-3-(3-dimethylaminopropyl) carbodiimide (EDC), as well as the difficulty in removing residual byproducts from the NHS/EDC reaction [39,40,41]. The incorporation of 3-APBA was confirmed by NMR and FTIR spectroscopy, while the degree of substitution was quantified using UV–Vis spectroscopy. Thermal properties of the modified polysaccharides were compared using TGA and DTG analyses. The resulting APBA-functionalized SG (SG-APBA) was subsequently crosslinked with TA via dynamic boronate ester bonds, leading to the formation of SG-APBA/TA (SAT) hydrogels. The rheological and morphological characteristics of the hydrogels were systematically evaluated, and their antioxidant and antibacterial activities were investigated. TA release profiles under different pH and glucose conditions were assessed, and cytocompatibility was confirmed using MTT assays with HEK 293 cells.

We hypothesized that grafting boronic acid groups onto SG would impart dynamic covalent bonding ability, enabling the formation of hydrogels with superior injectability, self-healing capacity, and multi-stimuli responsiveness. Furthermore, we anticipated that the synergistic incorporation of tannic acid would not only reinforce the hydrogel network through hydrogen bonding and boronate ester interactions but also endow the system with intrinsic antioxidant and antibacterial functionalities. Based on this hypothesis, we expected that the resulting boronic-acid-modified succinoglycan hydrogels would exhibit dual responsiveness to pH and glucose, leading to controlled tannic acid release while simultaneously providing bioactive antioxidant and antibacterial activities, thus establishing a multifunctional platform for advanced biomedical applications.

## 2. Results and Discussion

### 2.1. Characterizaion of SG-APBA

#### 2.1.1. Synthesis of SG-APBA and Calculation of Degree of Substitution

**Figure 1:**
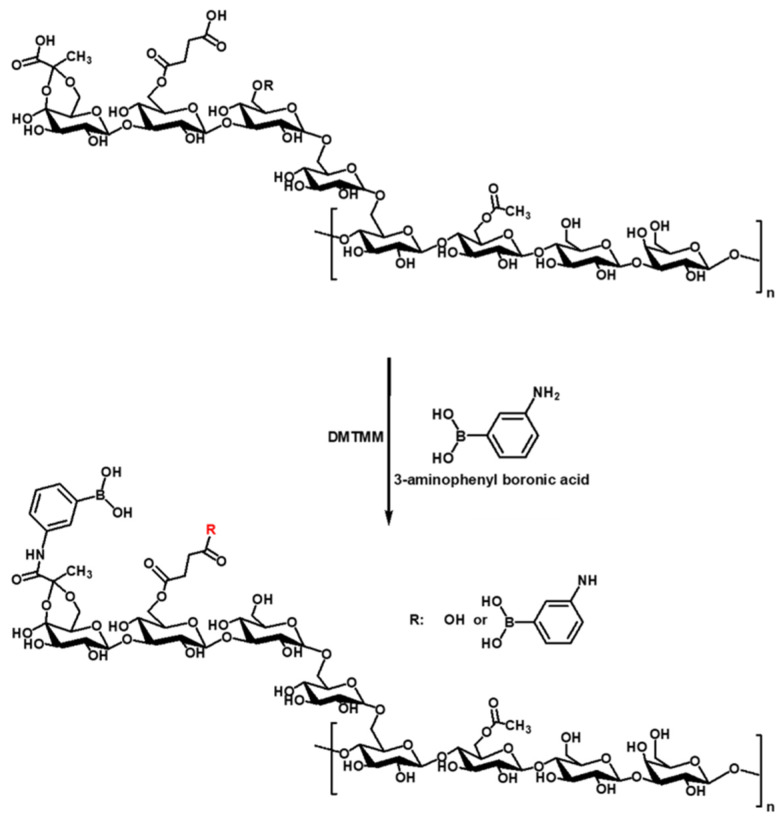
Schematic illustration of SG-APBA preparation.

Amidation of 3-APBA onto SG was mediated via 4-(4, 6-dimethoxy-triazine-2-yl)-4-methyl morpholine hydrochloride (DMTMM) reaction. Figure 1 shows the chemical structure after amidation. 3-APBA could be introduced with carboxylic group of pyruvate and succinate in SG.

After fixing the ratio of DMTMM, the ratio of APBA was adjusted and experiments were conducted under four different conditions to select the condition of optimized APBA incorporation. To assess the extent of APBA attachment, UV-absorbance measurements were performed [42].

**Figure 2:**
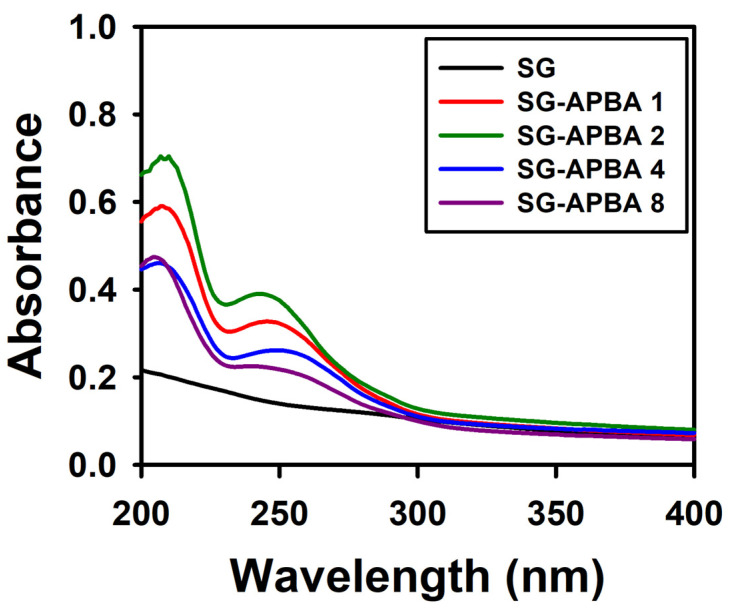
UV absorbance of SG-APBA derivatives.

Prior to assessing the degree of substitution, a calibration curve correlating the UV absorbance of APBA with its concentration was generated. The standard curve for APBA is shown in Figure S1, where absorbance values increased linearly as APBA concentration rose, thus enabling the construction of an accurate calibration model. The UV spectra of SG and its derivatives with attached APBA are presented in Figure 2. The most significant incorporation of APBA was observed in the SG-APBA 2 sample. Given that SG offers two potential sites for amidation, the attachment percentages of APBA at each condition were calculated based on the calibration curve, yielding values of 17.1%, 24.3%, 10.7%, and 4.24%. These calculations indicated that the condition corresponding to SG-APBA 2 produced the highest degree of substitution as determined by UV absorbance analysis.

#### 2.1.2. Rheological Analysis of SG-APBA

**Figure 3:**
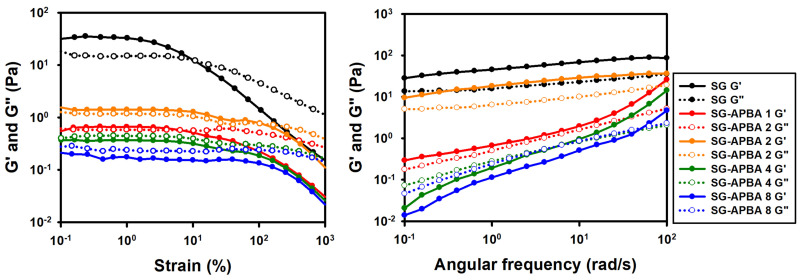
(**a**) Amplitude sweep and (**b**) frequency sweep test of SG and SG-APBA.

The rheological properties of SG and SG-APBA solutions were examined using strain amplitude sweep (Figure 3a) and frequency sweep tests (Figure 3b). After grafting of APBA, both G′ and G″ decreased compared to SG, although the overall G′ and G″ increased as the degree of substitution (DS) of SG-APBA increased. This reduction in G′ and G″ upon APBA incorporation was attributed to the presence of phenyl substituents, which disrupted hydrogen bonding and entanglements, thereby increasing free volume [43,44]. However, at higher DS, the increased number of boronic acid sites facilitated additional boronate ester bond formation, partially restoring G′ and G″ values [45]. Based on these findings, SG-APBA 2, which exhibited the highest degree of substitution along with favorable G′ and G″ values, was selected for further analysis.

#### 2.1.3. ^1^H NMR Analysis

**Figure 4:**
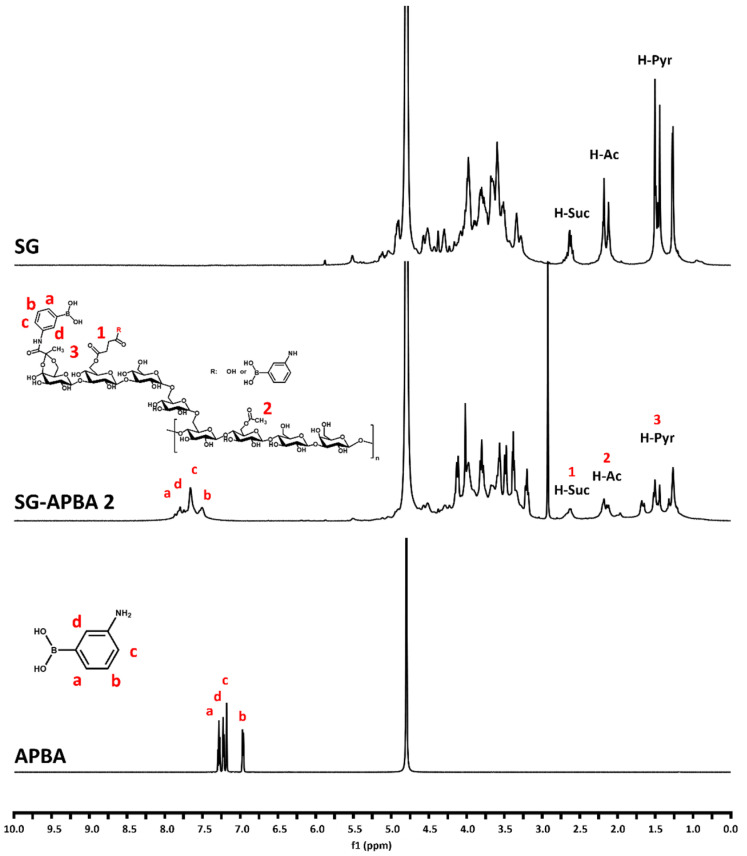
^1^H NMR spectra of SG, APBA and SG-APBA 2.

To verify the successful incorporation of APBA into SG, ^1^H NMR spectroscopy was conducted. The spectrum of SG-APBA is presented in Figure 4. Control samples of pure SG and APBA were also analyzed to identify spectral alterations resulting from the conjugation process. In the case of SG, characteristic peaks were observed at approximately 2.56, 2.22, and 1.57 ppm, corresponding to the succinyl, acetyl, and pyruvyl groups of SG, respectively. After amidation with APBA, new peaks appeared in the 7.0 to 8.0 ppm region, aligning with the chemical shifts associated with the aromatic protons of APBA. These observations were consistent with previously reported data on the amidation of polysaccharide–APBA conjugates, confirming the successful modification of SG with APBA [46,47].

#### 2.1.4. FTIR Analysis

**Figure 5:**
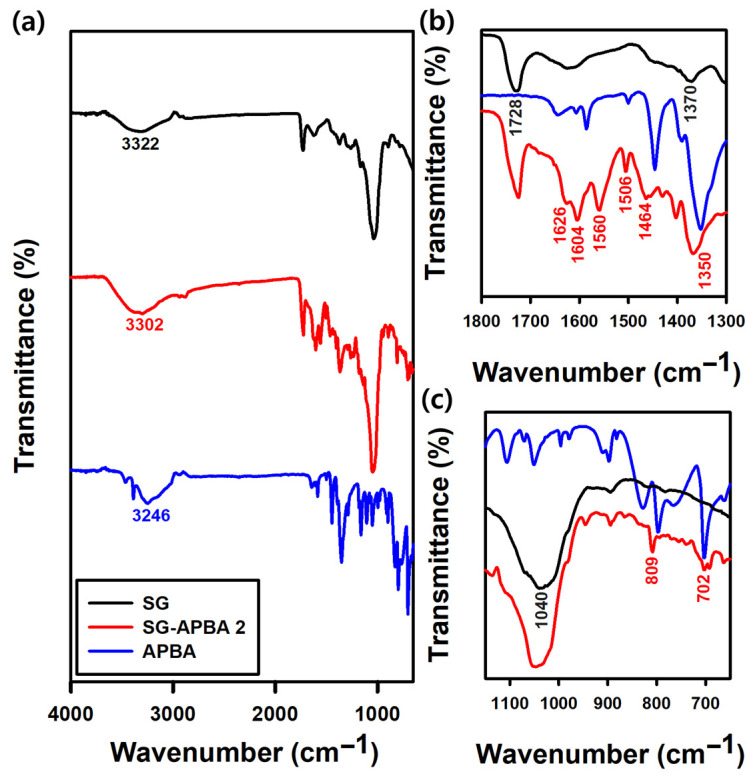
(**a**) FTIR spectra from 4000 to 650 cm^−1^ of SG, APBA and SG-APBA 2. (**b**) FTIR spectra from 1800 to 1300 cm^−1^ of SG, APBA and SG-APBA 2. (**c**) FTIR spectra from 1150 to 650 cm^−1^ of SG, APBA and SG-APBA 2.

Additional structural analysis was conducted using FTIR spectroscopy, and the resulting spectra are presented in Figure 5. The FTIR spectrum of SG displayed a broad peak at 3322 cm^−1^ corresponding to O–H stretching vibrations, while a peak at 1728 cm^−1^ was attributed to the C=O stretching vibration of the carboxyl groups. A peak at 1370 cm^−1^ was observed, which was assigned to the symmetric stretching vibration of the –COO^−^ group, characteristic of pyruvate and succinate residues. Moreover, a peak at 1040 cm^−1^ indicated the asymmetric C–O–C stretching vibration, representing the glycosidic linkages that form the backbone of the polysaccharide [48]. Compared with the spectra prior to amidation, new peaks emerged in the spectrum of SG-APBA 2 at 1626 cm^−1^ and 1560 cm^−1^, corresponding to the amide I and amide II bands, respectively. These peaks were associated with C=O stretching combined with N–H bending (amide I) and N–H bending coupled with C–N stretching (amide II), indicating the formation of amide bonds [28,46]. The introduction of boronic acid groups through APBA modification was confirmed by new peaks at 1464 cm^−1^ and 1350 cm^−1^, corresponding to the stretching vibrations of C–B and B–O bonds [49,50]. Further characteristic vibrations of the aromatic ring of APBA were observed at 1604 cm^−1^, 1506 cm^−1^, and 809 cm^−1^, while a distinct band at 702 cm^−1^ was attributed to out-of-plane C–H bending vibrations of the phenyl group [51,52,53].

#### 2.1.5. Thermal Analysis

**Figure 6:**
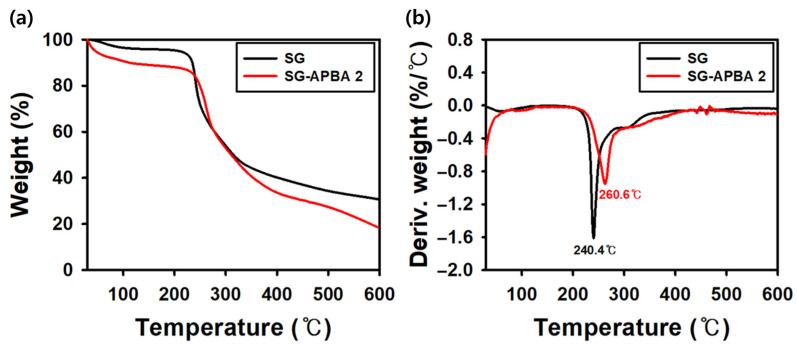
(**a**) TGA and (**b**) DTG curves of the SG and SG-APBA 2.

To assess and compare the thermal stability of SG and SG-APBA 2, thermogravimetric analysis (TGA) and derivative thermogravimetry (DTG) curves (Figure 6) were obtained over the temperature range of 30 °C to 600 °C. The initial slight weight loss observed in both samples between 30 °C and 210 °C was attributed to the desorption of water molecules bound through hydrogen bonds [54]. A more significant weight reduction occurred between 200 °C and 400 °C. Analysis of the DTG curves revealed that SG-APBA 2 experienced its maximum rate of weight loss at 260.6 °C, whereas SG reached this point at 240.4 °C. These results indicated that incorporating 3-aminophenylboronic acid into the structure enhanced the thermal stability of SG-APBA 2 by effectively delaying the onset of thermal decomposition [55].

#### 2.1.6. XRD Analysis

**Figure 7:**
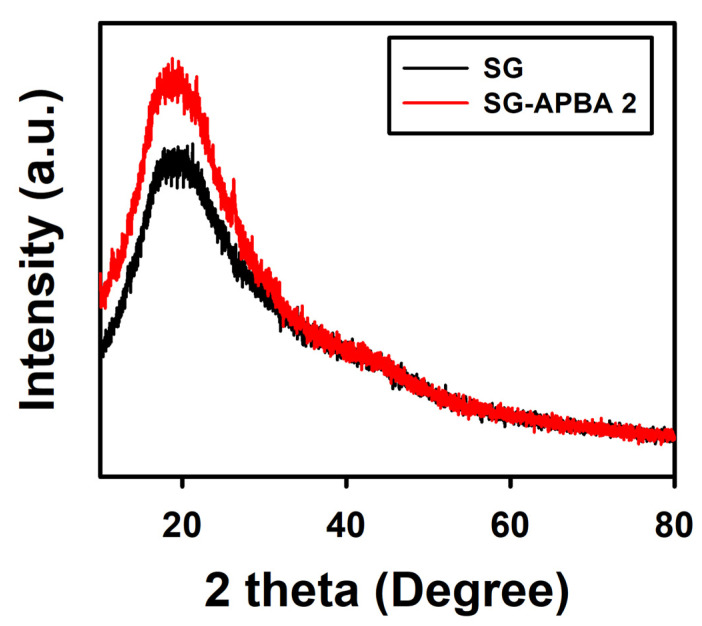
XRD patterns of the SG and SG-APBA 2.

X-ray diffraction (XRD) analysis was conducted to investigate the structural alterations in SG and SG-APBA 2, as depicted in Figure 7. The diffraction pattern for SG exhibited a broad peak at 2θ = 19.3°. In contrast, the XRD peak of SG-APBA 2 shifted slightly from 19.3° to 18.9°. This shift suggested that the incorporation of 3-aminophenylboronic acid moieties induced a structural rearrangement within SG [56]. The peak intensity increased following the introduction of APBA, indicating a rise in crystallinity. This enhancement was likely due to π-π stacking interactions among the phenylboronic acid groups [47].

#### 2.1.7. Viscosity Analysis of SG-APBA

**Figure 8:**
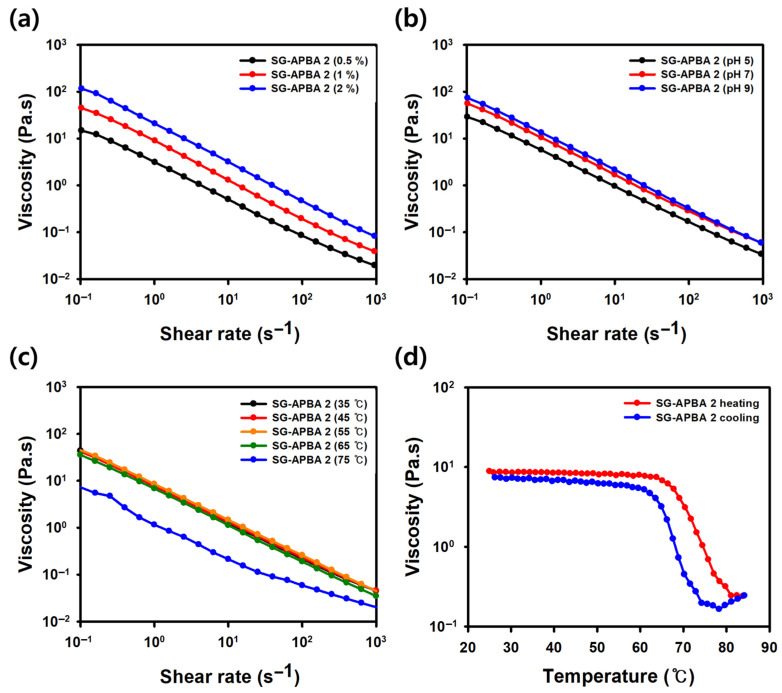
Viscosity of SG-APBA 2 (**a**) depending on concentration, (**b**) depending on pH, (**c**) depending on temperature and (**d**) temperature sweep test.

The viscosity of SG-APBA was measured under different concentrations, pH levels, and temperatures. For concentration dependence, solutions were prepared at 0.5%, 1%, and 2%, and their viscosity changes were evaluated (Figure 8a). The results showed that as concentration increased from 0.5% to 2%, viscosity rose from approximately 0.75 Pa·s to 3.2 Pa·s at a shear rate of 10 s^−1^, indicating that higher concentrations contributed to enhanced viscoelasticity [57,58]. Compared to SG (Figure S2a), SG-APBA exhibited lower viscosities at all concentrations, suggesting that the introduction of APBA reduced the overall physical properties. Regarding pH dependence, viscosity measurements were conducted at pH 5.0, 7.4, and 9.0. As shown in Figure S2b, SG displayed minimal variation in viscosity across this pH range. Conversely, as illustrated in Figure 8b, SG-APBA demonstrated a clear pH-responsive behavior with viscosity increasing at higher pH values. At a shear rate of 10 s^−1^, the viscosity increased from 1.0 Pa·s at pH 5.0 to 1.7 Pa·s at pH 7.4, and further to 2.2 Pa·s at pH 9.0. This trend confirms the formation of pH-sensitive boronate ester bonds, as higher pH facilitates more APBA–diol linkages, increasing crosslink density [45,59]. Temperature dependence was analyzed between 35 °C and 75 °C. As shown in Figure S2c, the viscosity of SG remained relatively stable up to 55 °C, indicating thermal stability within this range, but experienced a noticeable decline at 65 °C. Conversely, Figure 8c revealed that SG-APBA maintained a similar viscosity profile up to 65 °C, with values of approximately 1.3 Pa·s at 35 °C and 1.1 Pa·s at 65 °C at a shear rate of 10 s^−1^, but viscosity dropped sharply to 0.2 Pa·s at 75 °C. Viscosity measurements during heating and cooling cycles from 25 °C to 85 °C (with a rate of 10 °C/min and a shear rate of 1 s^−1^) showed that the viscosity of SG decreased at around 60 °C but recovered upon cooling (Figure S2d). SG-APBA exhibited a similar pattern, with a delayed viscosity drop occurring at approximately 66 °C (Figure 8d). These findings indicated that while the intermolecular interactions in SG-APBA weakened at elevated temperatures, the polymer maintained its viscosity and demonstrated enhanced thermal stability [60].

### 2.2. Preparation of SAT Hydrogels

**Figure 9:**
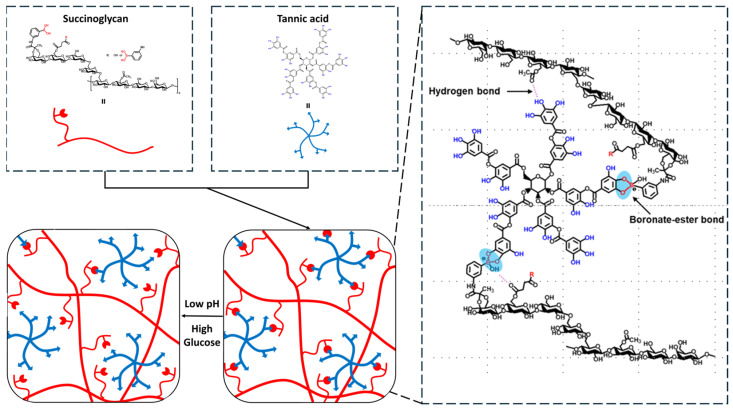
Scheme of SAT hydrogel network formation mechanism.

**Figure 10:**
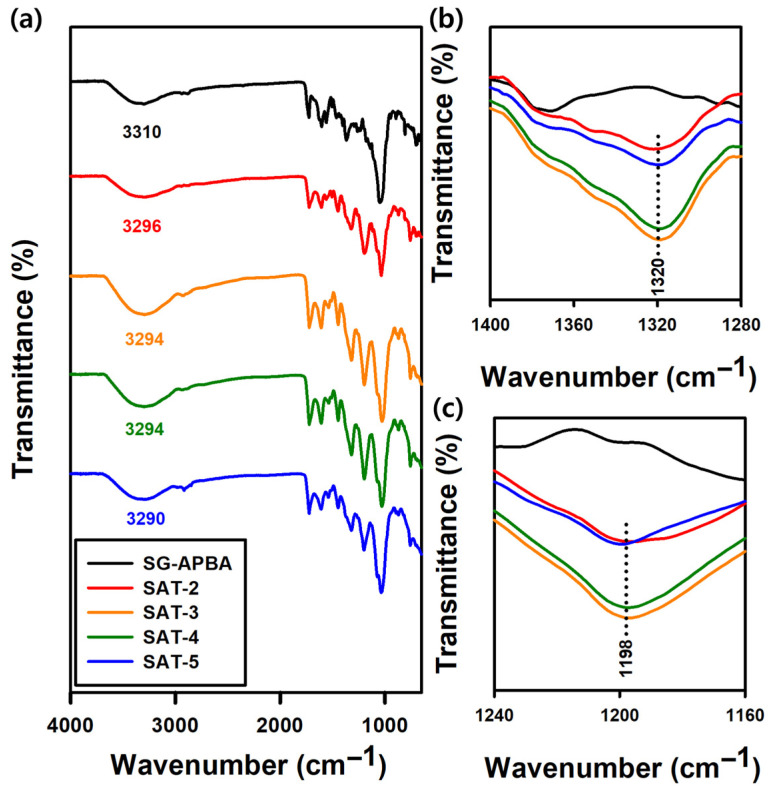
(**a**) FTIR spectra from 4000 to 650 cm^−1^ of SG-APBA 2 and SAT hydrogel. (**b**) FTIR spectra from 1400 to 1280 cm^−1^ of SG-APBA 2 and SAT hydrogel. (**c**) FTIR spectra from 1240 to 1160 cm^−1^ of SG-APBA 2 and SAT hydrogel.

The SAT hydrogels were synthesized by combining a solution of SG-APBA with TA. As depicted in Figure 9, the hydrogel network was formed through dynamic boronate ester bonds between the phenylboronic acid groups of SG-APBA and the catechol and galloyl groups of TA, with TA acting as a crosslinker [61]. Additionally, hydrogen bonds were established between TA and the carboxyl groups of SG-APBA. The impact of the APBA substitution degree on mechanical properties of the hydrogels was assessed using rheological analysis. The detailed composition of the hydrogels is described in Table S1, and the corresponding frequency sweep and strain amplitude sweep results are presented in Figure S3. The results revealed that the G’ increased with higher DS values, indicating that a higher DS results in a greater crosslinking density of the hydrogel network. SG-APBA 2/5% TA hydrogel exhibited the highest G’ value; therefore, SG-APBA 2 was selected as the base material for subsequent SAT hydrogel experiments. Furthermore, the effect of the SG-APBA 2 concentration on hydrogel properties was investigated. The successful formation of the SAT hydrogel was confirmed through FTIR analysis. As shown in Figure 10, the FTIR spectra revealed new peaks at 1320 cm^−1^ and 1198 cm^−1^, corresponding to B–O–C bonds, indicative of boronate ester formation [62,63]. Also, the broad hydroxyl stretching peaks shifted to around 3290–3296 cm^−1^, suggesting hydrogen bonding interactions between hydroxyl groups, the carboxyl groups of SG-APBA, and the phenolic groups of TA [64].

### 2.3. Rheological Analysis of SAT Hydrogels

#### 2.3.1. Frequency Sweep and Amplitude Sweep Test

**Figure 11:**
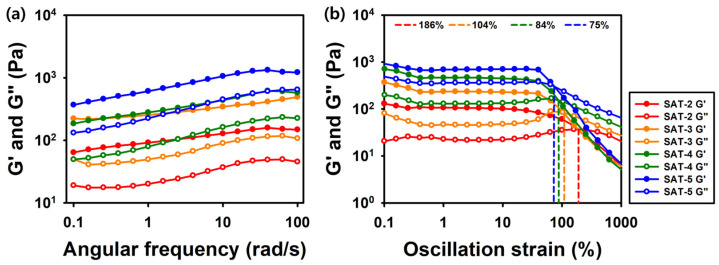
Rheological analysis of SAT hydrogels. (**a**) Frequency sweep test, strain = 1%, (**b**) Strain amplitude sweep test, frequency = 1.0 rad/s.

The oscillation angular frequency sweep test was conducted within the frequency range of 0.1 to 100 rad/s at a fixed shear strain of 1%. As shown in Figure 11a, G′ values consistently exceeded G″ values across all samples, confirming the elastic solid behavior of the hydrogels [65]. Furthermore, the G′ values for SAT-2, SAT-3, SAT-4, and SAT-5 were 148 Pa, 491 Pa, 574 Pa, and 1220 Pa, respectively. An increase in SG-APBA concentration corresponded with higher G′ values, which aligned with previous studies showing that increased crosslinking density enhances the mechanical strength of hydrogel networks [66]. The strain amplitude sweep test was conducted to assess the flexibility of the SAT hydrogels over a strain range of 0.1% to 1000% at a fixed frequency of 1.0 rad/s (Figure 11b). The crossover points of the storage modulus (G′) and loss modulus (G″) were observed at 186%, 104%, 84%, and 75% for SAT-2, SAT-3, SAT-4, and SAT-5, respectively, indicating the gel-to-sol transition of the hydrogels [67]. The results implied that a higher SG-APBA content caused the gel-to-sol transition to occur at lower strain values. This behavior is consistent with other dynamic covalent hydrogels reported a gel–sol transition at a strain of 117%, positioning our SAT hydrogels, particularly SAT-2, as having superior structural stability under high deformation [68].

#### 2.3.2. Viscosity Measurements

**Figure 12:**
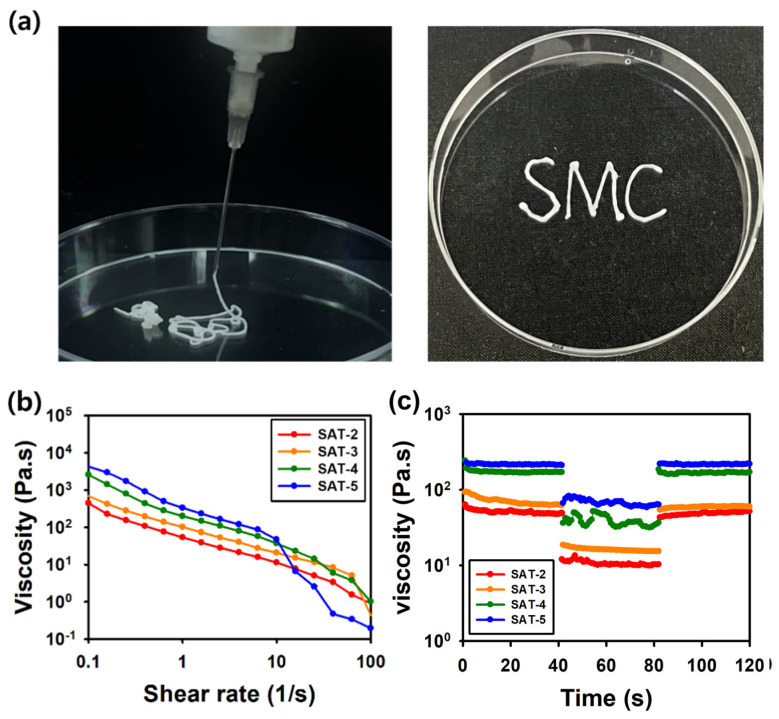
(**a**) Photographs demonstrating the injectable behavior, (**b**) Shear thinning behavior and (**c**) Thixotropic behavior of the SAT hydrogel.

As shown in Figure 12a, the SAT hydrogel passed through a syringe and reformed into a gel after injection. Figure 12b demonstrated that the hydrogel exhibited a reduction in viscosity as the shear rate increased within the range of 0.1 to 100 s^−1^. Under applied shear stress, the viscosity gradually diminished, allowing the hydrogel to flow through a needle. Additionally, the viscosity increased with higher SG-APBA content, indicating that increased crosslinking required higher shear rates to induce flow [69]. Moreover, the thixotropic behavior of the hydrogels was evaluated using a three-interval thixotropy test (3ITT) by alternating shear rates between 1 s^−1^ and 10 s^−1^. As shown in Figure 12c, the hydrogel rapidly recovered to initial viscosity when the high shear was removed. The extent of regeneration was quantified by comparing the viscosity during the third step to the initial rest viscosity in step one. The regeneration ratios were calculated to be 83.8%, 73.7%, 98.3%, and 98.8% for SAT-2, SAT-3, SAT-4, and SAT-5, respectively [70]. Collectively, these findings indicated that the SAT hydrogels exhibited both shear-thinning and thixotropic properties suitable for injectable applications [71].

#### 2.3.3. Alternative Step Strain Sweep Test

**Figure 13:**
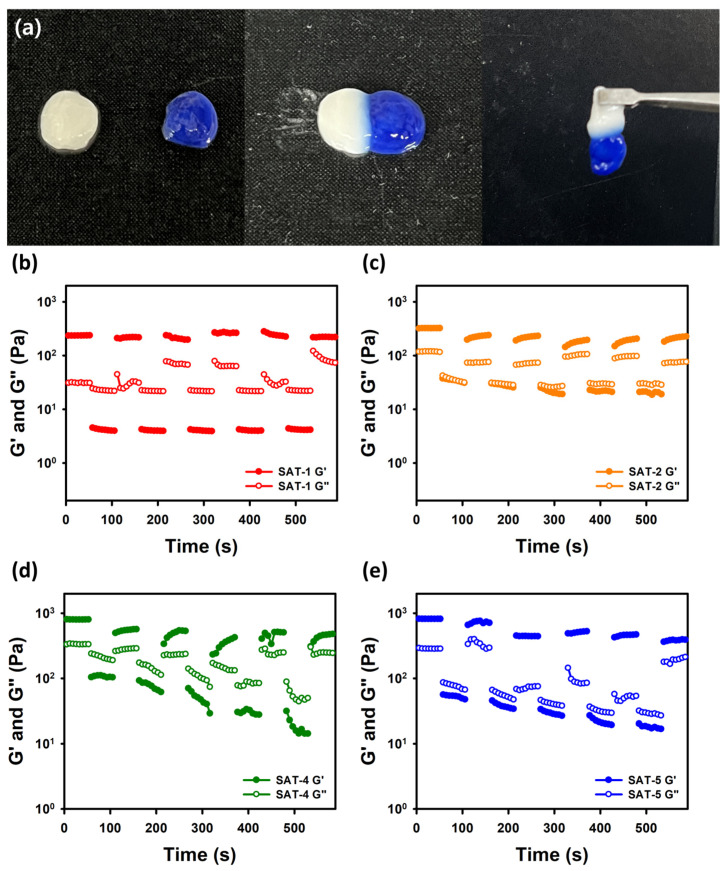
(**a**) Photographs of SAT hydrogel for self-healing property. (**b**) Rheological measurements with alternating step-strain sweep test of SAT-2, (**c**) SAT-3, (**d**) SAT-4 and (**e**) SAT-5.

The self-healing ability of the SAT hydrogels was evaluated through visual inspection and rheological analysis. As depicted in Figure 13a, two differently colored hydrogels were brought into contact and demonstrated effective adhesion. To further investigate their self-healing capacity, alternating step-strain sweep tests were conducted with the strain oscillating between 0.1% and 300% at a constant frequency of 10.0 rad/s (Figure 13b–e). The percentages of recovery for SAT-2, SAT-3, SAT-4, and SAT-5 were 93.4%, 65.0%, 53.5%, and 46.8%, respectively. This restoration was correlated to the formation of dynamic boronate ester bonds through the boric acid groups present in the hydrogel network [24]. However, the decrease in recovery efficiency from SAT-2 to SAT-5 was associated with the increasing concentration of SG-APBA [72].

### 2.4. Morphologies of SAT Hydrogels

**Figure 14:**
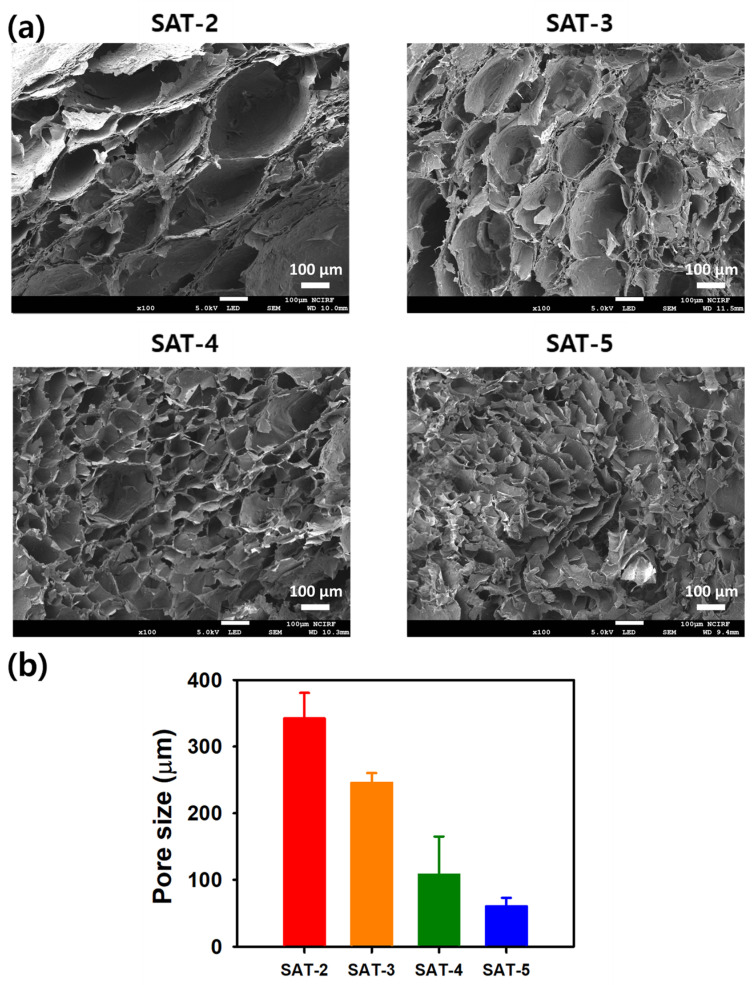
(**a**) Morphological analysis of SAT hydrogels. (**b**) Bar graph showing the pore size results based on SEM image.

The morphology of lyophilized SAT hydrogels was characterized using FE-SEM. As depicted in Figure 14, all samples displayed a porous network structure that originated from the formation of dynamic boronic ester crosslinks between the boronic acid groups of SG-APBA and TA. Analysis of the images indicated a correlation between the SG-APBA content and pore size; specifically, the SAT-2 hydrogel had the largest pore diameter at 342.2 μm, while the pore size decreased as the SG-APBA concentration increased. This pattern was consistent across all samples, with SAT-3 showing a pore diameter of 245.5 μm, SAT-4 measuring 107.6 μm, and SAT-5 having the smallest pore diameter of 60.3 μm. The decrease in pore size is likely due to a higher crosslinking density caused by the increased number of boronic acid groups, which form more extensive intermolecular crosslinks with TA [73,74]. The enhanced crosslinking density creates a more compact network structure, thereby reducing the void space and overall porosity of the hydrogel matrix. These findings demonstrate that the pore architecture of SAT hydrogels can be precisely controlled by modulating the SG-APBA concentration, providing a valuable strategy for tailoring the structural properties of these boronic ester-crosslinked systems for specific applications.

### 2.5. Antioxidant Activity

**Figure 15:**
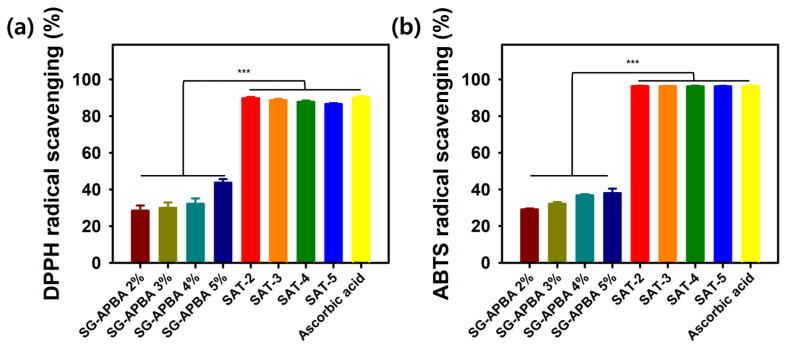
Antioxidant activity of the SAT hydrogels characterized by (**a**) DPPH radical scavenging and (**b**) ABTS radical scavenging method. Results are reported as mean ± standard deviation (SD), and statistical significance was determined by one-way ANOVA at a *p*-value of (*** < 0.005).

Excessive accumulation of reactive oxygen species (ROS) at wound sites has been known to induce oxidative stress, leading to cytotoxic damage to DNA and enzymatic functions. Incorporating antioxidant properties into wound dressings is considered a promising strategy to promote healing by scavenging ROS [75]. Hydrogels endowed with ROS-buffering capacity could potentially mitigate damage resulting from heightened inflammation and thereby facilitate tissue regeneration. Tannic acid (TA), a natural polyphenol abundant in plants such as green tea, grapes, and oak, exhibited potent antioxidant activity attributable to its multiple phenolic hydroxyl groups [76]. TA was utilized as a crosslinking agent, thereby imparting the SAT hydrogels with anticipated antioxidant functionalities. To assess these properties, the free radical scavenging abilities against DPPH and ABTS radicals were evaluated. As illustrated in Figure 15, the DPPH radical scavenging activity of SG-APBA increased proportionally with its content, showing values of 28.4%, 30.0%, 32.2%, and 43.8% at SG-APBA concentrations of 2%, 3%, 4%, and 5%, respectively. The corresponding SAT hydrogels, labeled SAT-2, SAT-3, SAT-4, and SAT-5, demonstrated DPPH scavenging percentages of 89.8%, 88.7%, 87.8%, and 86.6%. Similarly, in the ABTS radical scavenging assay, SG-APBA at 2% through 5% exhibited scavenging efficiencies of 29.1%, 32.2%, 36.8%, and 38.0%, respectively, while all SAT hydrogels achieved over 96% in scavenging activity. The precise numeric values for the antioxidant activity are presented in Table 1. This demonstrates the potent antioxidant contribution of tannic acid, as SAT hydrogels exhibit significantly higher radical scavenging capabilities compared to hydrogel systems based primarily on boronic acid derivatives, such as hyaluronic acid–phenylboronic acid (HA-PBA) hydrogels, which have reported hydroxyl radical scavenging effects of 67.72% [77]. These findings confirmed that the SAT hydrogels possessed robust free radical scavenging capabilities, which were expected to reduce oxidative stress and thereby enhance wound healing.

### 2.6. Antibacterial Activity

**Figure 16:**
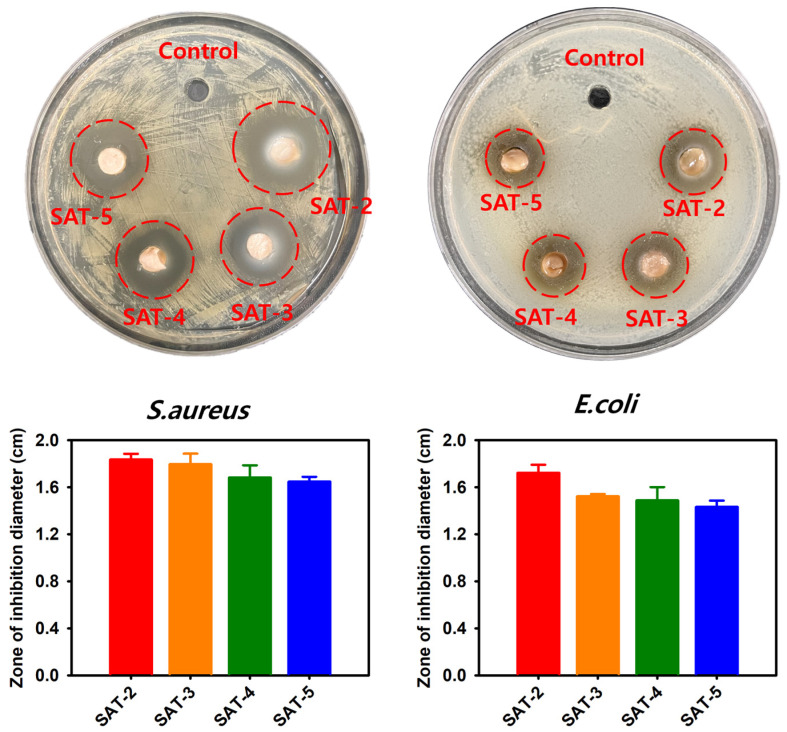
Antibacterial activities of the SAT hydrogels characterized by inhibition zone assay.

Bacterial infections can provoke persistent inflammation, which significantly disturbs the wound healing process [78]. Therefore, biomaterials with intrinsic antibacterial properties are desirable. TA has been recognized for its antibacterial activity, which operates through several mechanisms: (1) iron chelation activity and inhibiting iron uptake, (2) interference with cell wall synthesis, (3) disruption of cell membrane integrity [79]. *S. aureus* (Gram-positive) and *E. coli* (Gram-negative) are the most common bacteria associated with wound infections and are major obstacles to the wound healing process [80]. The antibacterial efficacy of the hydrogels was assessed via an inhibition zone assay against *S. aureus* and *E. coli*. As shown in Figure 16, all hydrogel samples produced larger zones of inhibition than the control group for both bacterial strains. The inhibition zones for *S. aureus* surrounding the SAT-2, SAT-3, SAT-4, and SAT-5 hydrogels measured 1.83 ± 0.05 cm, 1.80 ± 0.09 cm, 1.68 ± 0.10 cm, and 1.65 ± 0.04 cm, respectively. For *E. coli*, the inhibition zones of the same hydrogels were 1.72 ± 0.07 cm, 1.52 ± 0.02 cm, 1.49 ± 0.11 cm, and 1.43 ± 0.05 cm, respectively. Notably, SAT -2 demonstrated the most potent antibacterial activity, which was attributed to the faster release of TA resulting from its comparatively lower crosslinking density [72]. Due to its notable antibacterial performance, the degradation and TA release behaviors of SAT -2 hydrogel were further examined.

### 2.7. Dual-Responsive In Vitro Degradation Profile

**Figure 17:**
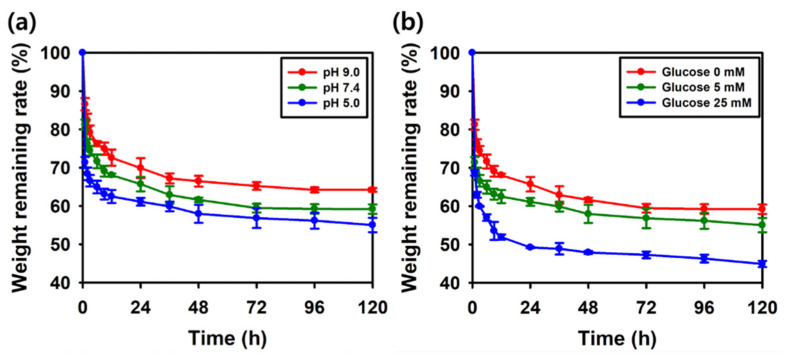
Degradation pattern of SAT-2 hydrogel under (**a**) different pH, (**b**) different glucose concentrations.

Boronic-acid-based materials are widely recognized for their stimuli-responsive properties, particularly in response to pH changes and molecules containing cis-diols [81]. This responsiveness is primarily attributed to the reversible formation and dissociation of boronate ester bonds between boronic acid groups and cis-diol functionalities, which enable dynamic network rearrangement under external stimuli [82]. Under acidic conditions, boronic acids predominantly exist in a trigonal planar form, resulting in ester bonds that are less stable and more prone to hydrolysis. Conversely, at alkaline pH, boronic acids become deprotonated to form tetrahedral boronate ions, which exhibit greater stability and have stronger binding affinity toward diols. These pH-dependent changes increase the overall crosslinking stability of the hydrogel network, thus slowing down its degradation, whereas acidic environments promote bond cleavage and accelerate degradation processes [83,84]. As shown in Figure 17a, the degradation profile of the SAT hydrogel demonstrated pH dependence, with degradation rates increasing as the pH decreased. In addition to pH sensitivity, boronic-acid-based hydrogels also exhibited glucose-responsive degradation due to the reversible and competitive binding of glucose to boronic acid moieties [85,86]. Glucose, a cis-diol-containing molecule, formed cyclic boronate esters with boronic acids, competing with the original diol-based crosslinkers. This competitive binding disrupted pre-existing boronate ester bonds, which reduced the crosslinking density and weakened the hydrogel network. As a result, hydrogels exposed to higher glucose concentrations showed accelerated network disassembly and fragmentation [87]. In Figure 17b, the degradation of the SAT hydrogels was observed to be concentration-dependent on glucose, with higher glucose levels leading to increased structural breakdown. These findings are consistent with the competitive binding mechanism, indicating that glucose can effectively trigger de-crosslinking within the boronate ester-crosslinked network of the SAT hydrogel.

### 2.8. Dual-Responsive TA Release Profile

**Figure 18:**
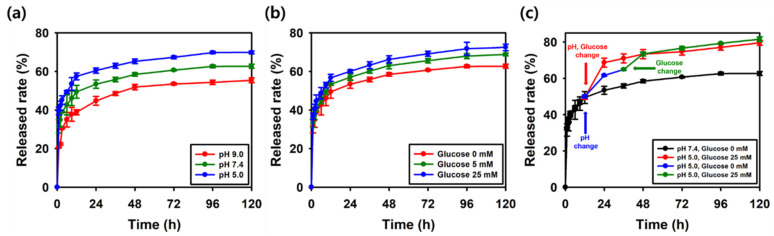
TA release pattern under (**a**) different pH, (**b**) different glucose concentrations, (**c**) pH change from 7.4 to 5.0 and glucose concentration change from 0 mM to 25 mM simultaneously and sequentially.

To assess the environmental responsiveness, the release of TA was monitored under varying pH levels and glucose concentrations. As shown in Figure 18a, the SAT-2 hydrogel displayed a pH-sensitive release pattern in PBS buffer at 37 °C. After 120 h, the total amount of TA released was 55.4% at pH 9.0, 62.7% at pH 7.4, and 69.9% at pH 5.0. These results are consistent with the decreased stability of boronate ester bonds in more acidic conditions [88]. Furthermore, in PBS buffer at pH 7.4 with different glucose levels (0, 5, and 25 mM), glucose-responsive release was observed (Figure 18b). After 120 h, the total TA released was 62.7% without glucose, increasing to 68.9% at 5 mM, and reaching 72.5% at 25 mM. This tendency can be explained by the competitive interaction between glucose molecules and boronic ester bonds [89]. To clearly illustrate the combined influence of pH and glucose levels, two additional experiments were conducted (Figure 18c). In the first, both pH and glucose concentration were simultaneously altered at 12 h (from pH 7.4 to 5.0 and from 0 mM to 25 mM glucose). This triggered a burst release, with TA release increasing from 53.4% at 24 h in the control condition (pH 7.4, 0 mM glucose) to 69.7%, and reaching 79.6% at 120 h. In the second experiment, the pH was shifted from 7.4 to 5.0 at 12 h, followed by an increase in glucose concentration from 0 mM to 25 mM at 36 h. Under these conditions, TA release was 61.6% at 24 h, then rose to 73.5% at 48 h after glucose addition, and ultimately reached 81.6% at 120 h. These findings demonstrate that SAT hydrogels respond synergistically to acidic pH and elevated glucose levels, enabling controlled and enhanced TA release in response to specific environmental cues. To provide molecular-level evidence for dual-responsive TA release pattern, the FTIR spectra of SAT-2 hydrogels after exposure to different environments are presented (pure, PBS, pH 5.0, glucose 25 mM, and combined pH 5.0 and glucose 25 mM). As shown in Figure S4, the characteristic B–O–C peaks at 1320 cm^−1^ and 1198 cm^−1^ were significantly decreased in intensity after exposure to pH and/or glucose-containing environments. This decrease confirms the cleavage of boronate ester linkages, which is attributed to the protonation of boronic acid groups and competitive diol exchange with glucose. A sustained release profile is critical for wound healing applications, and a release profile over 120 h is particularly advantageous for long-term therapeutic applications, contrasting with systems based on carboxymethyl chitosan–tannic acid, which exhibit a rapid burst release of nearly 60% within 3 h [90].

### 2.9. In Vitro Cytotoxicity of SAT Hydrogels

**Figure 19:**
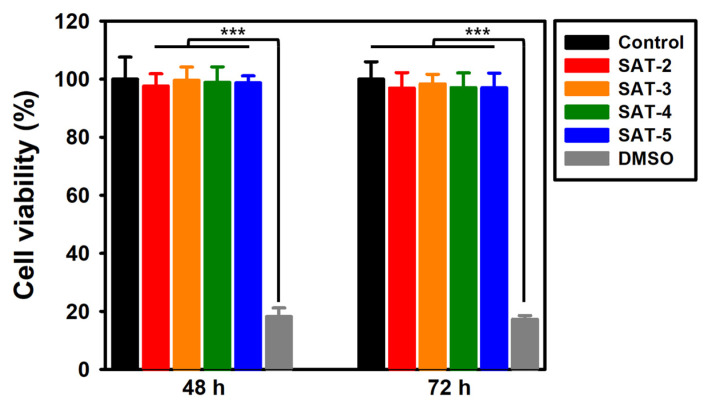
Cytotoxicity analysis of SAT hydrogels determined by MTT assay. Results are reported as mean ± standard deviation (SD), and statistical significance was determined by one-way ANOVA at a *p*-value of (*** < 0.005).

Biocompatibility of the SAT hydrogels was assessed with an MTT assay, using HEK 293 cells. Cell viability was evaluated by comparing groups treated with SAT hydrogels to a negative control group treated with DMEM and a positive control group treated with DMSO. As shown in Figure 19, after 48 h of incubation, the viability of cells treated with SAT-2, SAT-3, SAT-4, and SAT-5 was 97.59%, 99.53%, 98.82%, and 98.68%, respectively. After 72 h, the corresponding cell viability values were 96.91%, 98.35%, 97.06%, and 96.98%. Although HEK293 cells were used in this study to assess general cytocompatibility [91,92], additional cytotoxicity tests might be necessary depending on the specific application of the hydrogel. For instance, if the hydrogel was intended for wound healing purposes, conducting toxicity assessments with skin-relevant cell types such as fibroblasts and keratinocytes would provide more pertinent insights aligned with the intended use of the hydrogel [93].

## 3. Conclusions

SG-APBA was prepared by performing amidation of SG with DMTMM. When SG-APBA was combined with TA, it resulted in the formation of multi-responsive polysaccharide-based hydrogels. The addition of TA as a crosslinking agent improved the hydrogel’s stability through boronate ester and hydrogen bonding interactions, while also imparting antioxidant and antibacterial properties inherent to TA. Viscosity and rheological analyses demonstrated that the mechanical strength of the hydrogels increased with a higher SG-APBA content. The resulting SAT hydrogels were injectable, with self-antioxidant and self-antibacterial abilities, and exhibited tunable rheological properties and pore sizes depending on the SG-APBA concentration. Furthermore, the TA release pattern from SAT hydrogels was more pronounced under acidic pH (pH 5.0) compared to basic pH (pH 9.0), and it increased under high glucose conditions (25 mM) relative to glucose-free conditions. Notably, when both pH and glucose conditions were simultaneously varied, the hydrogels displayed a synergistic double-shock response, resulting in enhanced and controlled release of tannic acid compared to single-stimulus conditions. These findings confirm the dual pH and glucose responsiveness of the SAT hydrogels and highlight their potential as a multifunctional platform for controlled TA release with antioxidant and antibacterial activities. Moreover, the SAT hydrogel showed no cytotoxicity toward HEK-293 cells in vitro. Altogether, these self-healing, injectable and dual pH- and glucose-responsive multifunctional SAT hydrogels exhibit potential for biomedical applications, particularly in diabetic wound repair. However, future in vivo studies focusing on wound healing and tissue regeneration are required to validate their therapeutic potential for clinical implementation.

## 4. Materials and Methods

### 4.1. Reagents and Materials

The *Sinorhizobium meliloti* Rm1021 strain was obtained from the MCRB (Microbial Carbohydrate Resource Bank) at Konkuk University (Seoul, Republic of Korea). 2-(N-morpholino)ethanesulfonic acid buffer (MES), 3-Aminophenylboronic acid (APBA) (purity 98%), Tannic acid (TA), 2,2-diphenyl-1-Picrylhydrazyl (DPPH) (purity 98%) and iron (II) sulfate heptahydrate (FeSO_4_⋅7H_2_O) were purchased from Sigma Aldrich (Taufkirchen, Germany). 4-(4,6-Dimethoxy-1,3,5-triazin-2-yl)-4-methylmorpholinium tetrafluoroborate (DMTMM) (purity > 95%) was obtained from TCI (Tokyo, Japan). Potassium sodium tartrate (purity 99%) was purchased from Duksan (Ansan, Republic of Korea).

### 4.2. Culture Conditions and Isolation of Succinoglycan (SG)

The isolation and purification of SG from *Sinorhizobium meliloti* Rm1021 were performed based on a previously reported method [94]. The bacteria were cultured for 7 days at 30 °C with a shaking speed of 180 rpm in medium containing D-mannitol (10 g/L), glutamic acid (1 g/L), K_2_HPO_4_ (1 g/L), MgSO_4_·7H_2_O (0.2 g/L), and CaCl_2_·2H_2_O (0.04 g/L), which was adjusted to pH 7. After cultivation, the cells were pelleted by centrifugation (8000× *g*, 15 min, 4 °C), allowing for the collection of the supernatant. SG was precipitated by adding ethanol to the supernatant at a ratio of 1:3 (*v*/*v*). The resulting precipitate was re-dissolved in distilled water and subjected to dialysis (MWCO 12–14 kDa membrane) against distilled water for a period of 3 days for purification. After dialysis, the SG was freeze-dried for later application. To determine the molecular weight of SG, gel permeation chromatography (Waters, Milford, MA, USA) was conducted with 0.02 N sodium nitrate (0.8 mL/min, 30 °C) as a solvent. Pullulan was used as the calibration standard. The molecular weight (Mw) of SG was determined to be approximately 3.2 × 10^5^ Da based on gel permeation chromatography (GPC) analysis [95].

### 4.3. Preparation of 3-Aminophenylboronic Acid Grafted Succinoglycan (SG-APBA)

SG-APBA was synthesized by coupling APBA with SG in the presence of DMTMM as an activating reagent [96,97]. In brief, SG (1.0 g, 0.65 mmol) was dissolved in 90 mL of MES buffer (0.1 M, pH 6.0) and stirred at room temperature. Subsequently, a solution of DMTMM (689.6 mg, 2.5 mmol) was prepared in 5 mL of MES buffer and introduced into the SG solution, followed by stirring for 30 min to activate the carboxyl groups of SG. Afterward, APBA, dissolved in 5 mL of MES buffer, was added to the reaction mixture at various molar ratios relative to the carboxyl groups of SG, specifically at 1:1, 2:1, 4:1, and 8:1 [98]. The reaction proceeded at room temperature with stirring for 72 h. After the reaction, purification was carried out by dialyzing the mixture against distilled water through a 12–14 kDa MWCO membrane for 3 days. The dialyzed solutions were freeze-dried. To determine the degree of substitution (DS), UV absorbance at 295 nm was measured using a UV-Vis spectrophotometer (Shimadzu, Kyoto, Japan). A calibration curve was obtained from phenylboronic acid standards ranging from 0.0125 mg/mL to 0.1 mg/mL.

### 4.4. Characterizaion

#### 4.4.1. Nuclear Magnetic Resonance (NMR) Spectroscopy

1% of SG-APBA in D_2_O was prepared at 25 °C. The ^1^H NMR spectra were recorded in 600 MHz using a Bruker AvanceIII-600 spectrometer (Bruker, Karlsruhe, Germany).

#### 4.4.2. Fourier Transform Infrared (FTIR) Spectroscopy

FTIR analysis of the lyophilized samples were collected using an FTIR spectrometer (Spectrum Two, PerkinElmer, Waltham, MA, USA). The spectra were recorded within the range between 4000 and 650 cm^−1^ with a resolution of 2 cm^−1^.

#### 4.4.3. Thermal Gravimetric Analysis (TGA)

A Discovery TGA 5500 (TA Instruments, New Castle, DE, USA) was employed to analyze the thermal stability of SG and SG-APBA 2. Each sample (10 mg) was heated from 30 to 600 °C at 10 °C min^−1^ in a nitrogen environment.

#### 4.4.4. X-Ray Diffraction (XRD)

X-ray diffraction (XRD) patterns were recorded by an X-ray diffractometer (Rigaku SmartLab, Akishima, Japan) equipped with CuKα radiation operated at 40 kV and 30 mA. 2θ ranges were recorded within 20–80°.

### 4.5. Preparation of SG-APBA/TA (SAT) Hydrogels

SAT hydrogels were produced by combining solutions of SG-APBA and TA. In brief, stock solutions of SG-APBA at the required concentrations and a 5% (*w*/*v*) TA solution were prepared fresh. The SG-APBA and TA solutions were then mixed in a 4:1 (*v*/*v*) ratio at room temperature. Hydrogels designated as SAT-2, SAT-3, SAT-4, and SAT-5 were created using different concentrations of SG-APBA, specifically 2%, 3%, 4%, and 5% (*w*/*v*), respectively.

### 4.6. Rheological Analysis of SG and SG-APBA

Rheological measurements of SG and SG-APBA solutions were investigated with a DHR-2 rheometer (TA Instruments, New Castle, DE, USA). The samples, prepared in distilled water, were analyzed at 25 °C (except for temperature-dependent studies) with 60 mm parallel plates.

Amplitude sweep tests were conducted for SG and SG-APBA solutions within a strain range of 0.1 to 1000% at a fixed angular frequency of 1 rad/s. Frequency sweep tests were measured within range of 0.01 to 100 rad/s at a 1.0% constant strain. Viscosity tests of SG and SG-APBA 2 solution were performed at a shear rate of 0.1 to 1000 s^−1^. Measurements were conducted at polymer concentrations of 0.5, 1.0 and 2.0%. Except for the concentration-dependent tests, a 1% solution was used to evaluate the effects of pH and temperature on viscosity. The pH-dependent measurements were obtained at pH 5, 7, and 9, while the temperature-dependent tests were performed at 35, 45, 55, 65, and 75 °C. In case of the sweep test about temperature, the temperature was increased from 25 °C to 85 °C with a heating rate of 10 °C/min.

### 4.7. Rheological Properties of SAT Hydrogel

Rheological measurements of each SAT hydrogel were assessed by Rheometer DHR2 (TA Instruments, New Castle, DE, USA). The measurements were performed at room temperature utilizing a 20 mm parallel plate. Frequency sweep tests were performed by fixing 1.0% constant strain with the range of 0.1 and 100 rad/s. The amplitude sweep tests were measured over a range of 0.1 to 1000% and conducted under the same conditions. Viscosity analysis of SAT hydrogels was measured under a shear rate increasing from 0.1 to 100 s^−1^. Thixotropy was determined by alternating the shear rate from 1 s^−1^ to 10 s^−1^ for 40 s. Strain amplitude sweep test was measured under 10.0 rad/s by alternating the strain between 0.1% and 300% for 50 s.

### 4.8. Morphological Analysis of SAT Hydrogels

After lyophilization, the dried SAT hydrogels were sliced, affixed to double-sided carbon tape, and coated with a thin gold layer in a vacuum environment to facilitate conductivity. Their cross-sectional structures were then examined using a JSM-7800F Prime field-emission scanning electron microscope (JEOL, Akishima, Japan). The images were recorded at magnification of 100× with an excitation voltage of 5 kV.

### 4.9. Biological Activity of SAT Hydrogel

#### 4.9.1. Antioxidant Activities of SAT Hydrogel

DPPH and ABTS assays were used to determine the antioxidant capacity of the SAT hydrogel [99,100]. The concentration of 100 μM DPPH working solution was prepared. For the ABTS solution, 2.45 mM potassium persulfate and 7.00 mM ABTS was reacted for 24 h at room temperature in the dark. The reacted solution was diluted with PBS (pH 7.4) until the absorbance at 734 nm reaches a value of 0.70 ± 0.02. For antioxidant evaluation, 500 μL of each SAT hydrogel sample was put in 3 mL of radical working solution and incubated for 30 min for DPPH and 10 min for ABTS at 37 °C with dark conditions. The absorbance values of reacted mixtures were checked at 517 nm for DPPH and 734 nm for ABTS.

(1) $$\begin{array}{c} Scavenging\;activity\;\left(\%\right)=\frac{A_{control}-A_{sample}}{A_{control}}\times 100 \end{array}$$

*A_control_* represents the absorbance of the ABTS/DPPH radical working solutions, and *A_sample_* indicates the absorbance of the solution following sample incubation. Each test was performed five times to ensure accuracy

#### 4.9.2. Antibacterial Activity of SAT Hydrogel

Inhibition zone assay against *E. coli* (ATCC 25922) and *S. aureus* (ATCC 25923) was selected to assess the antibacterial activity of SAT hydrogel [101,102]. Each bacterial strain was pre-cultured in Luria–Bertani (LB) broth for 12 h. After cultivation, 100 μL of bacterial suspension was spread evenly onto an agar plate. SAT hydrogel samples (250 μL) were loaded on the agar plate. The plates were maintained at 37 °C for 18 h and then the inhibition zones were observed. Each experiment was conducted in triplicate.

### 4.10. In Vitro Hydrogel Degradation

Hydrogel degradation was studied in PBS at 37 °C with shaking at 100 rpm [103,104]. The initial sample weights were recorded prior to incubation. SAT hydrogels were then soaked in 10 mL of PBS at different values of pH (5.0, 7.4 and 9.0). At a predetermined time, the SAT hydrogels were weighed after wiping with filter paper.

(2) $$Weight\;remaining\;\left(\%\right)=\frac{W_{t}}{W_{0}}\times 100\;$$

*W*_0_ represents the initial weight of the hydrogels, while *W_t_* denotes the sample weight at a given time point.

The same procedure was applied to determine the degradation behavior of the hydrogels in different glucose concentrations (5 and 25 mM). All experiments were performed in triplicate.

### 4.11. TA Release Study

The TA release rate was analyzed via the tartaric acid colorimetric method [23,105]. 600 μL of SAT hydrogels was immersed in 10 mL of PBS solution (pH 5.0, 7.4, or 9.0) and incubated at 37 °C under shaking. 1 mL of SAT hydrogel extract was collected and then replenished with 1 mL of fresh PBS solution.

To prepare the detection reagent, 100 mg of FeSO_4_ and 500 mg of potassium sodium tartrate were dissolved in 100 mL of distilled water and react for 10 min at room temperature. TA standard solutions and hydrogel extract were prepared. Mix 100 μL of each standard solution or hydrogel extract with 50 μL of the detection reagent in 96-well plate. Incubate the mixture for 2 min at 37 °C and then add 20 μL of PBS solution. The absorbance was measured at 540 nm. The obtained calibration curve was then used to determine the TA concentration and release rate.

(3) $$TA\;release\;rate\;\left(\%\right)=\frac{C_{t}\times V_{1}+\sum \left(C_{t}\times V_{t}\right)}{W_{0}}\times 100\;\;\;$$

*C_t_* indicates the concentration of TA released at a given time point, calculated using the standard curve, *V*_1_ represents the 10 mL of total volume, *V_t_* refers to 1 mL of sampling volume, and *W*_0_ denotes the initial mass of TA in the SAT hydrogels. The same experimental procedure was conducted at different glucose levels (5 and 25 mM). All experiments were performed in triplicate.

### 4.12. In Vitro Cytotoxicity Test

The cytotoxicity of the hydrogel was evaluated via MTT assay [106]. Human embryonic kidney 293 (HEK-293) cells line was supplied by KCLB (Korean Cell Line Bank, Seoul, Republic of Korea). Cells were seeded in 96-well plates and maintained for 24 h at 37 °C in a 5% CO_2_ humidified incubator. The medium was then replaced with hydrogel extract-containing medium (200 μg/mL) for experimental group. The negative control consisted of untreated DMEM, whereas the positive control was prepared by supplementing DMEM with 10% DMSO. Following incubation periods of 48 and 72 h, MTT reagent was added to each well. Then, absorbance at 540 nm was measured to determine the cell viability.

(4) $$Cell\;viability\;\left(\%\right)=\frac{Absorbance\;of\;cells\;with\;samples}{Absorbance\;of\;negative\;control\;cells}\times 100\;$$

### 4.13. Statistical Analysis

Statistical analyses were performed using SigmaPlot 10.0 (Systat Software, San Jose, CA, USA). Results are reported as mean ± standard deviation (SD), and statistical significance was determined by one-way ANOVA at a *p*-value of (*** < 0.005).

## Figures and Tables

**Table 1:** Antioxidant activity of SG-APBA and SAT hydrogels.

Sample	DPPH	ABTS
SG-APBA 2%	28.4 ± 2.9	29.1 ± 0.5
SG-APBA 3%	30.0 ± 2.8	32.2 ± 1.0
SG-APBA 4%	32.2 ± 3.0	36.8 ± 0.7
SG-APBA 5%	43.8 ± 1.9	38.0 ± 2.4
SAT-2	89.8 ± 0.7	96.4 ± 0.2
SAT-3	88.7 ± 0.7	96.3 ± 0.2
SAT-4	87.8 ± 0.5	96.3 ± 0.3
SAT-5	86.6 ± 0.8	96.3 ± 0.3

## Data Availability

The original contributions presented in this study are included in the article. Further inquiries can be directed to the corresponding authors.

## Supplementary Materials

The following supporting information can be downloaded at: https://www.mdpi.com/article/10.3390/gels11110897/s1, Figure S1: UV absorbance standard curve for APBA. Figure S2. Viscosity of SG (a) depending on concentration, (b) depending on pH, (c) depending on temperature and (d) temperature sweep test. Figure S3. Rheological analysis of SG-APBA/5% TA hydrogels prepared with different degrees of APBA substitution. (a) Frequency sweep test, strain = 1%, (b) Strain amplitude sweep test, frequency = 1.0 rad/s. Figure S4. FTIR spectra of SAT-2 hydrogel in different conditions. Table S1. Preparation of SAT hydrogels with different degrees of APBA substitution.

## Abbreviations

The following abbreviations are used in this manuscript:

3-APBA	3-aminophenylboronic acid
DMTMM	4-(4,6-dimethoxy-triazine-2-yl)-4-methyl morpholine hydrochloride
DTG	Derivative thermogravimetry
EDC	1-ethyl-3-(3-dimethylaminopropyl) carbodiimide
G′	Storage modulus
G″	Loss modulus
LVR	Linear viscoelastic region
PBA	Phenylboronic acid
ROS	Reactive oxygen species
SAT	SG-APBA/TA hydrogel
SG	Succinoglycan
SG-APBA	APBA functionalized SG
TA	Tannic acid
TGA	Thermogravimetric analysis
XRD	X-ray diffraction
