# Supplementary material for: Injectable and Self-Healing Boronic-Acid-Modified Succinoglycan Hydrogels: Dual-Stimuli-Responsive Platforms for Controlled Tannic Acid Release
Source: Gels. 2025 Nov 9;11(11):897. doi: 10.3390/gels11110897 (PMC12652636; doi:10.3390/gels11110897)
Supplement: Supplementary file 1 [file gels-11-00897-s001.zip › gels-3938433-supplementary.pdf]

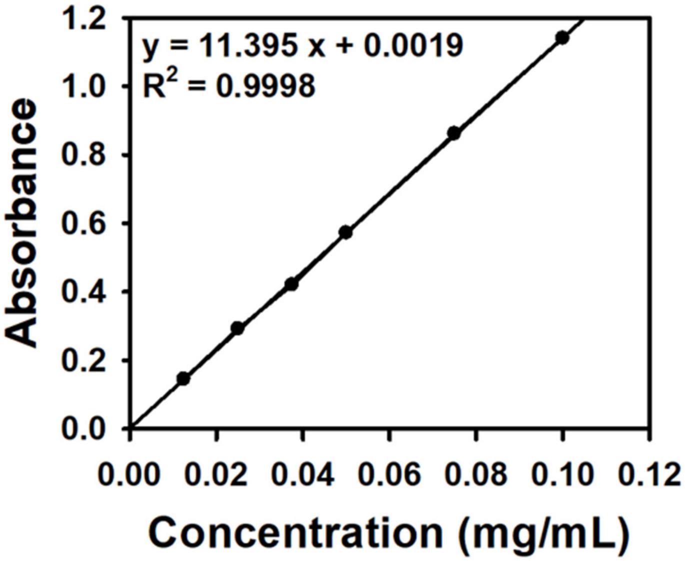

Figure S1. UV absorbance standard curve for APBA.

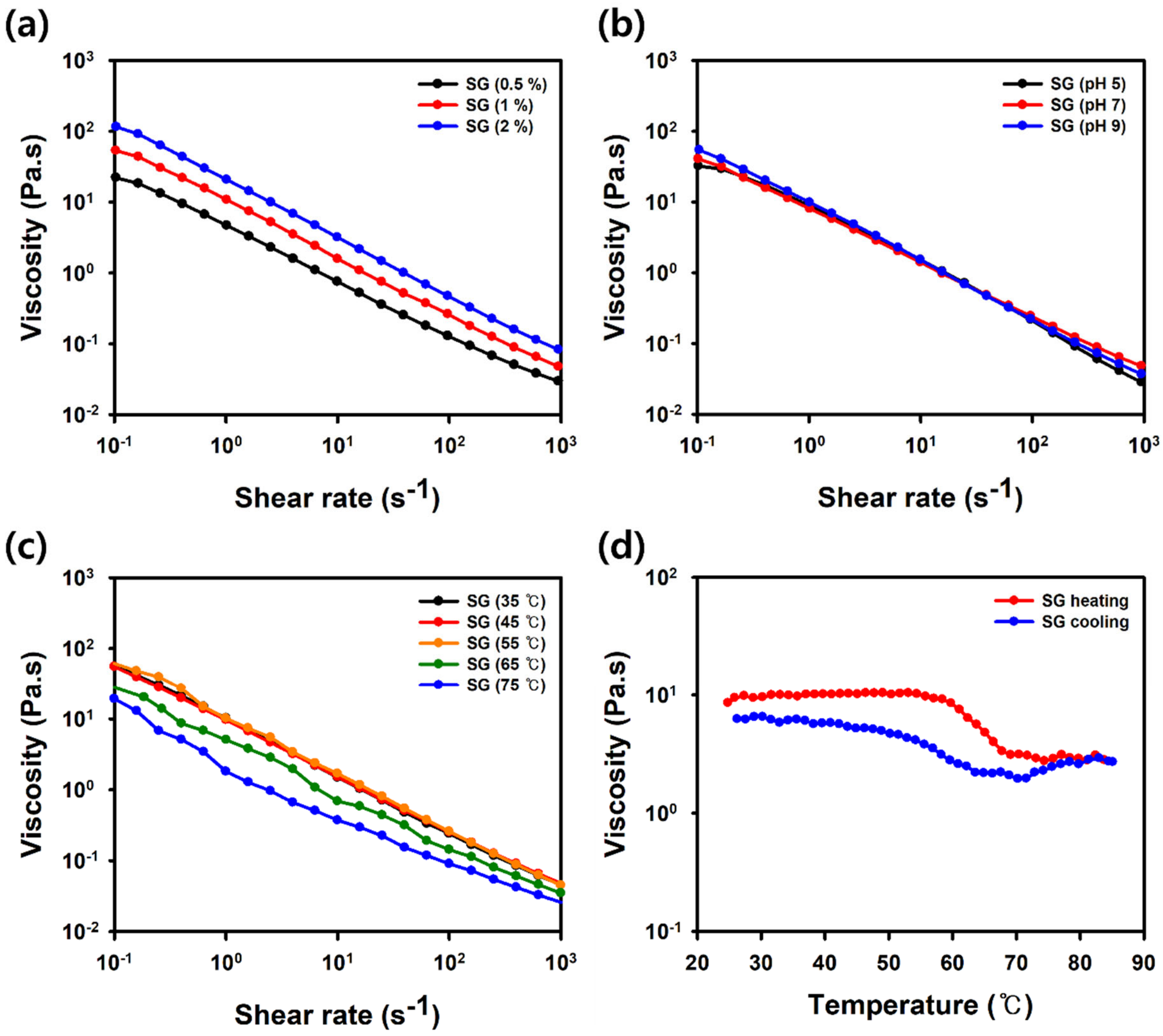

Figure S2. Viscosity of SG (a) depending on concentration, (b) depending on pH, (c) depending on temperature and (d) temperature sweep test.

Table S1. Preparation of SAT hydrogels with different degrees of APBA substitution.

Sample	DS (%)	SG-APBA concentration (w/v%)	TA concentration (w/v%)	SG-APBA:TA (v/v)
SG-APBA 1 / 5% TA	17.1	2.0	5.0	4:1
SG-APBA 2 / 5% TA	24.3	2.0	5.0	4:1
SG-APBA 3 / 5% TA	10.7	2.0	5.0	4:1
SG-APBA 4 / 5% TA	4.24	2.0	5.0	4:1

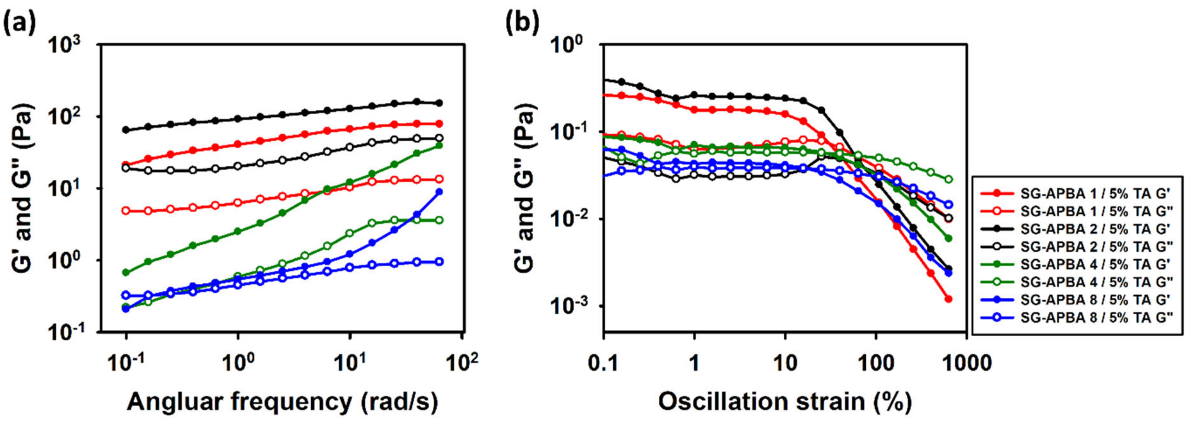

Figure S3. Rheological analysis of SG-APBA / 5% TA hydrogels prepared with different degrees of APBA substitution. (a) Frequency sweep test, strain = 1%, (b) Strain amplitude sweep test, frequency = 1.0 rad/s.

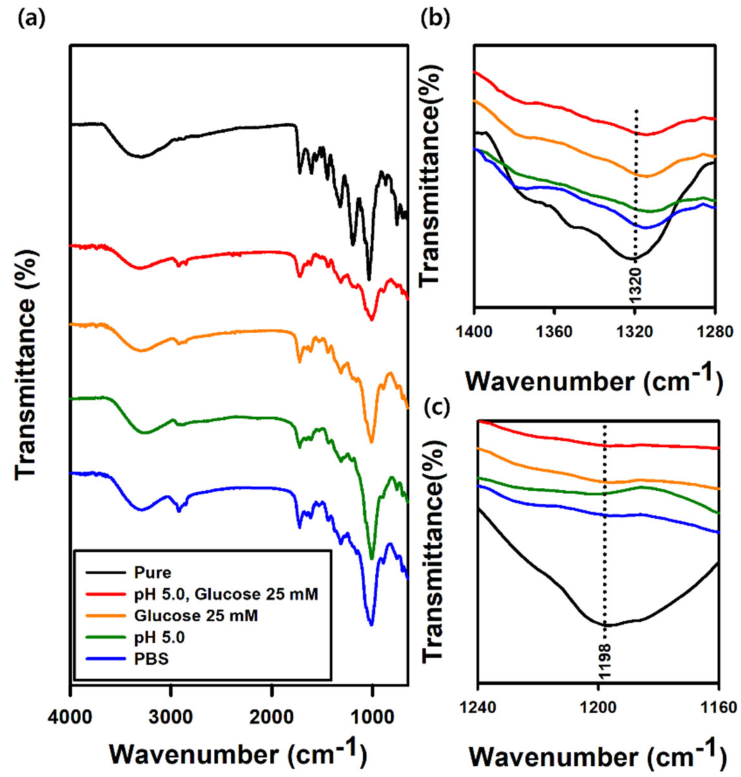

Figure S4. FTIR spectra of SAT-2 hydrogel in different conditions.
